# Establishing a New Platform to Investigate the Efficacy of Oncolytic Virotherapy in a Human Ex Vivo Peritoneal Carcinomatosis Model

**DOI:** 10.3390/v15020363

**Published:** 2023-01-27

**Authors:** Jana Koch, Julia Beil, Susanne Berchtold, Dina Mönch, Annika Maaß, Irina Smirnow, Andrea Schenk, Mary E. Carter, Linus D. Kloker, Tobias Leibold, Philipp Renner, Marc-H. Dahlke, Ulrich M. Lauer

**Affiliations:** 1 Dr. Margarete Fischer-Bosch Institute of Clinical Pharmacology, 70376 Stuttgart, Germany; 2 University of Tübingen, 72074 Tübingen, Germany; 3 Department of Medical Oncology and Pneumology, Virotherapy Center Tübingen (VCT), Medical University Hospital, 72076 Tübingen, Germany; 4 German Cancer Consortium (DKTK), German Cancer Research Center (DKFZ), 72076 Tübingen, Germany; 5 Department of General and Visceral Surgery, Robert Bosch Hospital, 70376 Stuttgart, Germany; 6 University Medical Centre Regensburg, 93053 Regensburg, Germany

**Keywords:** peritoneal carcinomatosis, ex vivo peritoneal culture model, virotherapy, oncolytic measles vaccine virus, oncolytic vaccinia virus

## Abstract

Oncolytic virotherapy constitutes a promising treatment option for many solid cancers, including peritoneal carcinomatosis (PC), which still represents a terminal stage of many types of tumors. To date, the in vitro efficacy of oncolytic viruses is mostly tested in 2D-cultured tumor cell lines due to the lack of realistic 3D in vitro tumor models. We have investigated the feasibility of virotherapy as a treatment option for PC in a human ex vivo peritoneum co-culture model. Human HT-29 cancer cells stably expressing marker genes GFP and firefly luciferase (GFP/luc) were cultured on human peritoneum and infected with two prototypic oncolytic viruses (GLV-0b347 and MeV-DsRed). Both viral constructs were able to infect HT-29 cells in patient-derived peritoneum with high tumor specificity. Over time, both GFP signal and luciferase activity decreased substantially, thereby indicating successful virus-induced oncolysis. Furthermore, immunohistochemistry stainings showed specific virotherapeutic infections of HT-29 cells and effective tumor cell lysis in infected co-cultures. Thus, the PC model established here provides a clinically relevant screening platform to evaluate the therapeutic efficacy of virotherapeutic compounds and also to investigate, in an autologous setting, the immunostimulatory potential of oncolytic viruses for PC in a unique human model system superior to standard 2D in vitro models.

## 1. Introduction

The peritoneum is the largest serous membrane of the human body, lining the internal organs, the abdominal wall, and the pelvis [1]. The large surface area of roughly 2 m^2^ as well as the permanently circulating peritoneal fluid make the peritoneum one of the organs most frequently affected by metastases of various tumor types, especially of gastrointestinal and gynecologic origins [2]. Once the diagnosis of peritoneal carcinomatosis (PC) is made, curative therapy is no longer possible in most cases and the prospects of any palliative therapy are very limited. Currently, multiple novel experimental therapies for solid tumors are being developed, including virotherapeutics, immunotherapies, novel antibodies, and innovative small molecule compounds; however, they are not yet clinically licensed for the treatment of PC.

The principle of oncolytic virotherapy relies on replication-competent viral vectors that selectively infect tumor cells, then replicate rapidly, and subsequently destroy the tumor cells (so-called oncolysis) while releasing thousands of new progeny viral particles [3]. It has been shown that the virus-induced tumor cell lysis triggers a systemic antitumoral immune reaction, which is the key success factor of this biological form of cancer therapy [4]. 

Thus, oncolytic virotherapy constitutes an upcoming treatment option for many solid cancers, including PC. However, the in vitro efficacy of oncolytic viruses so far can only be tested in 2D-cultured cell lines, as there is a lack of more realistic 3D in vitro tumor models to provide predictive preclinical results for PC patients [5]. Thus far, neither adequate in vitro nor human ex vivo models exist for PC. Several 3D-cultured organoids/spheroids or tissue slices of several tumor entities are currently exploited in the context of preclinical oncolytic virotherapy [5]. Hence, the establishment of peritoneal ex vivo models is of particular relevance not only for cancer research, including the study of treatment options for PC in a physiologically adequate tumor microenvironment but also for the development of personalized virotherapy in the future. Recently, a human ex vivo model was established to mimic PC formation and to test possible treatment options for PC [6,7].

In this study, we investigated the feasibility of oncolytic virotherapy as a treatment option for PC in a human ex vivo peritoneum co-culture model. In order to exploit a wide range of detection capabilities of this system, patient-derived healthy/nontumorous peritoneal tissue was co-cultivated with human HT-29 colorectal cancer cells, stably expressing marker genes GFP and firefly luciferase. These PC models were then infected with one of two prototypic oncolytic viruses from two different viral families. GLV-0b347 is a vaccinia virus (VACV) that belongs to the poxvirus family and has many advantages over other oncolytic vectors. It is a double-stranded DNA virus that can replicate extremely efficiently in the cytoplasm and can produce up to 5000 progeny virions per cell within 20 to 40 h. In addition, VACV is able to selectively infect and subsequently lyse almost any tumor type, thus being a highly versatile virotherapeutic agent [8,9]. The VACV construct GLV-0b347 has the unique feature of encoding the far-red-fluorescent marker protein TurboFP635, thereby allowing the in vitro detection of the successful infection of tumor cells with fluorescence microscopy [10]. The second oncolytic viral construct used in this work was the measles vaccine virus MeV-DsRed, which belongs to the paramyxovirus family. In general, MeV constructs used for virotherapy are derived from strains that have been utilized for years as vaccines and are therefore known to have an excellent safety profile. MeV-DsRed encodes the red-fluorescent marker protein DsRed, which also enables tracking of successful tumor cell infection [11]. 

Moreover, the double-labeled GFP/luc-positive HT-29 cells not only allow visual documentation of therapeutic success via GFP expression, but they also provide a direct quantification of the remaining (living) tumor cell population on the peritoneal tissue through the measurement of luciferase activity. These features, combined with the ability to track successful infection with oncolytic viruses based on marker gene expression, make the model system presented here a clinically relevant platform for the investigation of PC therapy.

## 2. Materials and Methods

### 2.1. Cell Culture

The human GFP/Luciferase (luc) dual-labeled HT-29 cancer cell line was purchased from GeneCopoeia^TM^ (Cat. SCL-C06-HLG, Rockville, MD, USA) and authenticated by short tandem repeat analysis (Eurofins, Ebersberg, Germany). Prior to use in experiments, GFP/luc HT-29 cells were tested for mycoplasma by polymerase chain reaction (PCR) using a Venor^®^GeM Classic kit (11-1025; Minerva Biolabs, Berlin, Germany) according to the manufacturer’s instructions. Cells were cultivated in McCoy’s 5A Medium with L-glutamine (Pan Biotech GmbH, Aidenbach, Germany) supplemented with 1% penicillin/streptomycin, 10% fetal calf serum (FCS) and with 1 μg/mL puromycin (Thermo Fisher Scientific, Waltham, MA, USA) for maintenance of selection in a 5% CO_2_ incubator at 37 °C.

### 2.2. Preparation and Culture of Peritoneal Tissue

Fresh peritoneal tissue was obtained directly from the operating room, with informed consent from the patients, and transferred immediately to the laboratory in E199 medium (Biochrom AG, Berlin, Germany). The tissue samples were then incubated for 15 min in phosphate-buffered saline (PBS) containing penicillin/streptomycin and amphotericin B (Sigma-Aldrich Chemie, St. Louis, MO, USA). Extraperitoneal fat was removed, and 7 × 7 mm tissue pieces were inserted between two stainless steel rings, which are available in various sizes, and thus enable large variations in the setting of these experiments. Moreover, stainless steel rings are more stable than plastic rings. In this setup, the ring system allows for tissue orientation with the mesothelial cells pointing upwards, thereby better reflecting the peritoneal cavity in the in vivo setting. The peritoneum was cultured in E199 medium containing penicillin/streptomycin, L-glutamine (Biochrom AG), FCS (Thermo Fisher Scientific), hydrocortisone (Sigma-Aldrich Chemie), fibroblast growth factor (PeproTech GmbH, Hamburg, Germany), and heparin (Biochrom AG), as described previously [12]. 

### 2.3. Preparation of Ex Vivo Peritoneum Co-Culture Model

Peritoneal tissue samples were inserted between stainless steel rings (internal diameter 4.4 mm). For co-culture, 2 × 10^5^ immortalized GFP/luc-labeled human HT-29 colorectal cancer cells were seeded onto the peritoneum and cultured in peritoneum medium for the indicated times in a 5% CO_2_ incubator at 37 °C.

### 2.4. Oncolytic Viral Constructs

The vaccinia virus (VACV) construct GLV-0b347 used in our studies was kindly provided by the Genelux Corporation (San Diego, CA, USA). In GLV-0b347 derived from the Western Reserve (WR) strain of vaccinia, the gene locus for thymidine kinase (J2R) was deleted by the insertion of the gene encoding for the far-red-fluorescent protein TurboFP635 under control of a vaccinia synthetic early/late promoter (P_SEL_) (Supplementary Materials Figure S1A).

The second oncolytic viral construct used was the measles vaccine virus MeV-DsRed, a vector derived from the measles Edmonston B vaccine strain, which carries the red-fluorescent marker gene *DsRed* in the leader position next to the *N* gene (Supplementary Materials Figure S1B). 

### 2.5. Infection of GFP/luc–HT-29 Cells in Cell Culture or in Ex Vivo Peritoneum Co-Culture Model

GFP/luc–HT-29 cells in 24-well plates were infected with GLV-0b347 or MeV-DsRed at different multiplicities of infection (MOI) in 250 μL of DMEM + 2% FCS (GLV-0b347) or OptiMEM (MeV-DsRed) (Invitrogen, Darmstadt, Germany). At 1 (GLV-0b347) or 3 (MeV-DsRed) hours postinfection (hpi), the inoculum was removed and a growth medium was added to the cells. At 72 and 96 hpi, the remaining tumor cell mass was analyzed using a sulforhodamine B (SRB) cell viability assay, via the measurement of luciferase activity, or by fluorescence intensity. Furthermore, fluorescence images were taken using a Leica DMi8 fluorescence microscope equipped with a DMC 4500 camera (Leica Biosystems, Wetzlar, Germany).

Ex vivo peritoneum co-culture models were cultured for 2–4 days. Then, infections with 1.5 × 10^6^ pfu (plaque-forming units) of GLV-0b347 or MeV-DsRed were performed in 50 µL of peritoneum medium without FCS. At 1 (GLV-0b347) or 3 (MeV-DsRed) hpi, the inoculum was removed and peritoneum medium with 20% FCS was added to the cells. Infected co-culture models were observed for 7 days postinfection (dpi), and at 2, 4, and 7 dpi, luciferase activity was measured and fluorescent images were taken using a Leica MZ10F stereo-fluorescence microscope (Leica Biosystems). 

### 2.6. Sulforhodamine B (SRB) Cell Viability Assay

As described previously [13], cells were washed with ice-cold PBS at 72 hpi, fixed with cold 10% trichloroacetic acid (TCA), and incubated at 4 °C for 30 min. After removing the TCA and drying, 0.4% SRB (Sigma-Aldrich, Taufkirchen, Germany) dissolved in 1% acetic acid was added, and cells were stained at room temperature (RT) for 10 min. After washing with 1% acetic acid and drying, 10 mmol/L Tris base (pH 10.5) was added to extract the protein-bound dye, and the cells were then incubated at RT for 10 min. Optical density was measured in a microtiter plate reader (Synergy HT, BioTek Instruments GmbH, Bad Friedrichshall, Germany) at a wavelength of 550 nm (reference wavelength at 620 nm).

### 2.7. Measurement of Luciferase Activity of GFP/luc–HT-29 Cells in Cell Culture or in Ex Vivo Peritoneum Co-Culture Models

The luciferase activity of GFP/luc–HT-29 cells in 24-well plates was measured in triplicate using the Bright-Glo^™^ Luciferase Assay System (Promega, Madison, WI, USA) according to the manufacturer’s instructions. HT-29 cells in 75 µL of medium were lysed with the addition of 75 µL of Bright-Glo^™^ Reagent and incubation for 2 min. Cell lysates were transferred into white 96-well plates, and the luminescence was measured with a Synergy HT reader (BioTek Instruments GmbH, Bad Friedrichshall, Germany).

For the measurement of luciferase activity in ex vivo peritoneum co-culture models, peritoneal tissue samples were removed from the steel rings and transferred into a 96-well plate with 70 µL of peritoneum medium. Cells were lysed by the addition of 70 µL of Bright-Glo^™^ Reagent and incubated for 5 min. Again, cell lysates were transferred into white 96-well plates and the luminescence was measured with a Synergy HT reader.

### 2.8. Immunohistochemistry

Formalin-fixed, paraffin-embedded sections (4 µm) of peritoneal tissue were stained with Mayer’s hematoxylin (Sigma-Aldrich Chemie) and eosin (Merck Chemicals GmbH, Darmstadt, Germany). For virus-specific antibody staining, the following antibodies and pretreatments were used: Measles (NP cl. 120 hybridoma cells, 1:1.000, heat-induced epitope retrieval at pH 6, ECACC Hybridoma Collection); Vaccinia (ab35219, 1:2.000, Abcam, Cambridge, United Kingdom). After antibody-specific epitope retrieval (Pronase 1 g/L, 107433, Merck Chemicals GmbH), endogenous peroxidase blocking (Dako, S2023) was performed for 10 min at RT. Primary antibody staining was performed at 4 °C overnight followed by peroxidase/DAB+-based detection using the Dako REAL EnVision Detection System (Dako, K7005). After proteinase K treatment, epithelial cell adhesion molecule (EpCAM) antibody staining (1:50, 248M-96, Cell Marque, Rocklin, CA, USA) was performed for 25 min on a Lab Vision Autostainer (Thermo Fisher Scientific GmbH, Waltham, MA, USA).

### 2.9. Statistical Analysis

Statistical analysis was performed with GraphPad Prism Version 9 (GraphPad Software Inc., San Diego, CA, USA). Treated samples from cell culture experiments without the co-culture of patient-derived peritoneum were compared to MOCK-treated control groups and the *p*-values were calculated using ordinary one-way ANOVA and Dunnett’s multiple comparison tests. In co-culture experiments regarding luciferase measurements, the significance between the two treatment groups was analyzed using unpaired *t*-tests. Four different *p*-values were determined: *p* < 0.05 (*), *p* < 0.01 (**), *p* < 0.001 (***), and *p* < 0.0001 (****).

## 3. Results

### 3.1. Virotherapeutic Treatment of GFP/luc-Labeled Human HT-29 Tumor Cells in Cell Culture

We initially investigated the efficacies of oncolytic vaccinia and measles viruses in a selected cancer cell line without the addition of patient-derived peritoneum.

**Figure 1:**
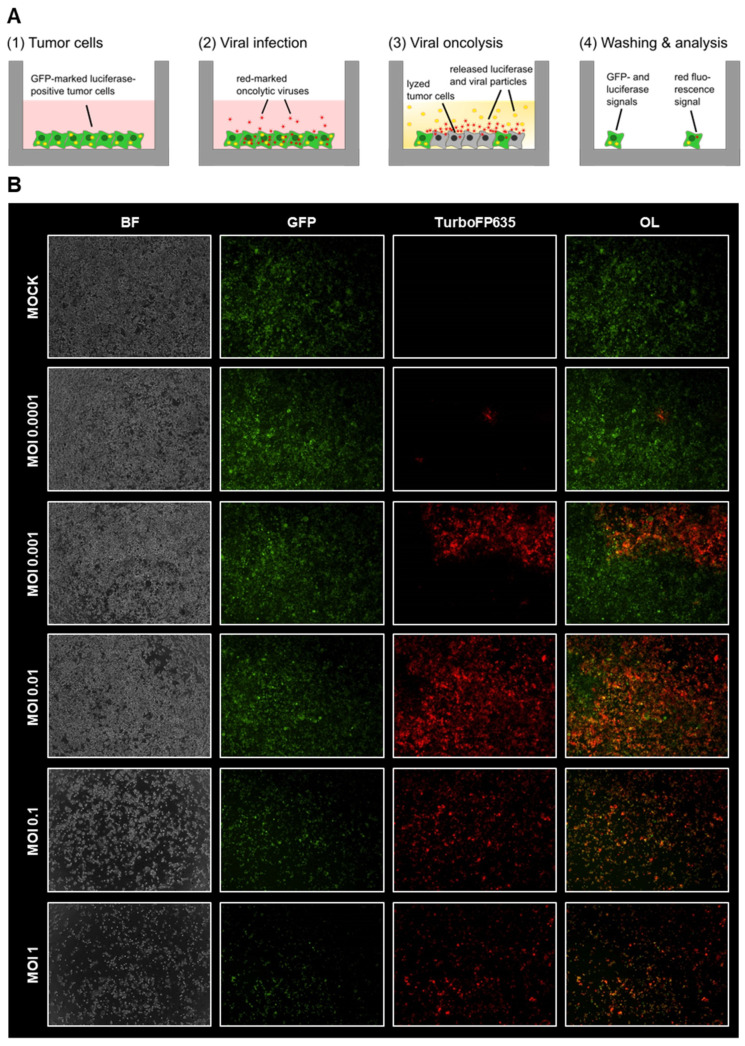
Virotherapeutic treatment of GFP/luc-labeled human HT-29 tumor cells in cell culture with GLV-0b347. (**A**) Schematic illustration of the three-step virotherapeutic process and associated detection capabilities: (1) GFP/luc-labeled HT-29 tumor cells were seeded into a 24-well cell culture plate. Successful plating of the cells can be verified by the determination of GFP fluorescence. (2) The treatment of HT-29 cells with oncolytic viruses encoding a red-fluorescent marker protein. The successful infection of the tumor cells can be verified by the determination of red fluorescence. (3)/(4) The viral oncolysis can be determined by a decrease in GFP as well as in luciferase activity. Over time, enhanced red fluorescence indicates an increasing number of tumor cells being infected by the red-fluorescence marker gene encoding virotherapeutic compounds. (**B**) The fluorescence images of HT-29 GFP/luc-labeled cells at 72 h postinfection (hpi) with GLV-0b347 at different multiplicities of infection (MOIs), as depicted. BF, brightfield; OL, overlay of GFP and TurboFP635 signal.

In a first approach, GFP/luc-labeled human HT-29 colorectal cancer cells were infected in vitro with increasing MOIs of vaccinia virus GLV-0b347 or measles vaccine virus MeV-DsRed, both labeled with a red-fluorescent marker protein in a complementary way to the green fluorescent HT-29 tumor cells. This sophisticated combination allows not only the visual documentation of successful viral infection, replication (red fluorescence), and oncolysis (green fluorescence) via fluorescence microscopy but also the tracking of therapeutic success by direct quantification of the residual living tumor cells through the measurement of remaining luciferase activity (Figure 1A).

**Figure 2:**
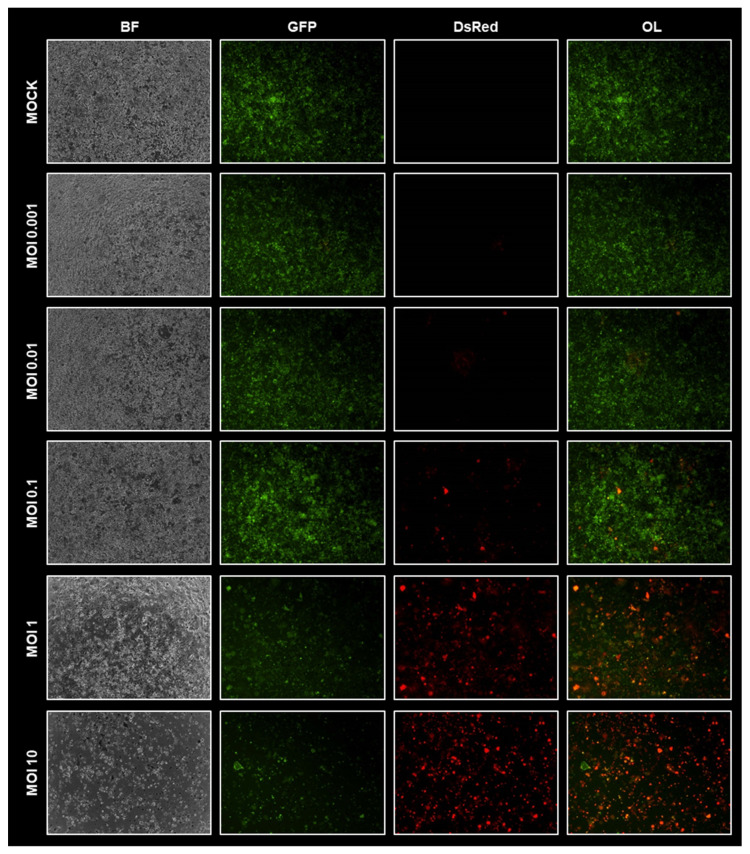
Virotherapeutic treatment of GFP/luc-labeled human HT-29 tumor cells in cell culture with MeV-DsRed. Fluorescence images of HT-29 GFP/luc-labeled cells at 72 h postinfection (hpi) with MeV-DsRed at different multiplicities of infection (MOIs), as depicted. BF, brightfield; OL, overlay of GFP and DsRed signal.

Fluorescence microscopy images taken at 72 hpi showed that GFP/luc–HT-29 cells could be successfully infected by both GLV-0b347 (Figure 1B) and MeV-DsRed (Figure 2) in an MOI-dependent manner. In tumor cells infected with GLV-0b347, an increasing fluorescent signal of the marker protein TurboFP635 was detected until a maximum was reached at an MOI of 0.01. At higher MOIs of 0.1 and 1, the fluorescent signals of TurboFP635 and GFP were significantly reduced, thereby indicating that oncolysis had already occurred substantially at this stage of infection after 72 h (Figure 1B). 

In GFP/luc–HT-29 cells infected by MeV-DsRed, a maximum of marker protein DsRed expression was reached at the highest examined MOI of 10, whereas GFP signaling was found to already decrease at MOIs of 1 and 10, an observation that indicates the onset of profound oncolysis. Furthermore, tumor cell death was confirmed in the corresponding transmitted light images (see buckling and detachment of the respective tumor cells) (Figure 2).

### 3.2. Comparison of Different Detection Options for the Oncolytic Activity of Virotherapeutic Compounds

Besides the possibility of visually detecting successful virus-mediated oncolysis by a reduction in the GFP signal in the fluorescence microscope, the sophisticated and thus state-of-the-art system used here allowed further analytical methods to prove the success of our tumoricidal therapy. Firstly, apart from the visual observation of either GFP or TurboFP635/DsRed fluorescence, the direct quantification of activity is possible by the determination of the expression of luciferase. This is because the human HT-29 cancer cell line used here is labeled not only with GFP but also with luciferase, which opens the possibility of quantifying the viability of remaining cells after virotherapy by the measurement of luciferase activity. Secondly, since the general susceptibility of cancer cells to virotherapeutic treatment with GLV-0b347 or MeV-DsRed alone, without co-culture on peritoneal tissue, was investigated here, it was also possible to evaluate the efficacy with a standardized cytotoxicity assay (e.g., with the sulforhodamine B (SRB) assay, which is the standard assay of the US National Cancer Institute to screen all kinds of cytotoxic substances [13]).

First, we investigated and compared the sensitivity of the different detection approaches to select the best analytical method for the measurement of the virotherapeutic efficacy in the ex vivo peritoneal co-culture model.

**Figure 3:**
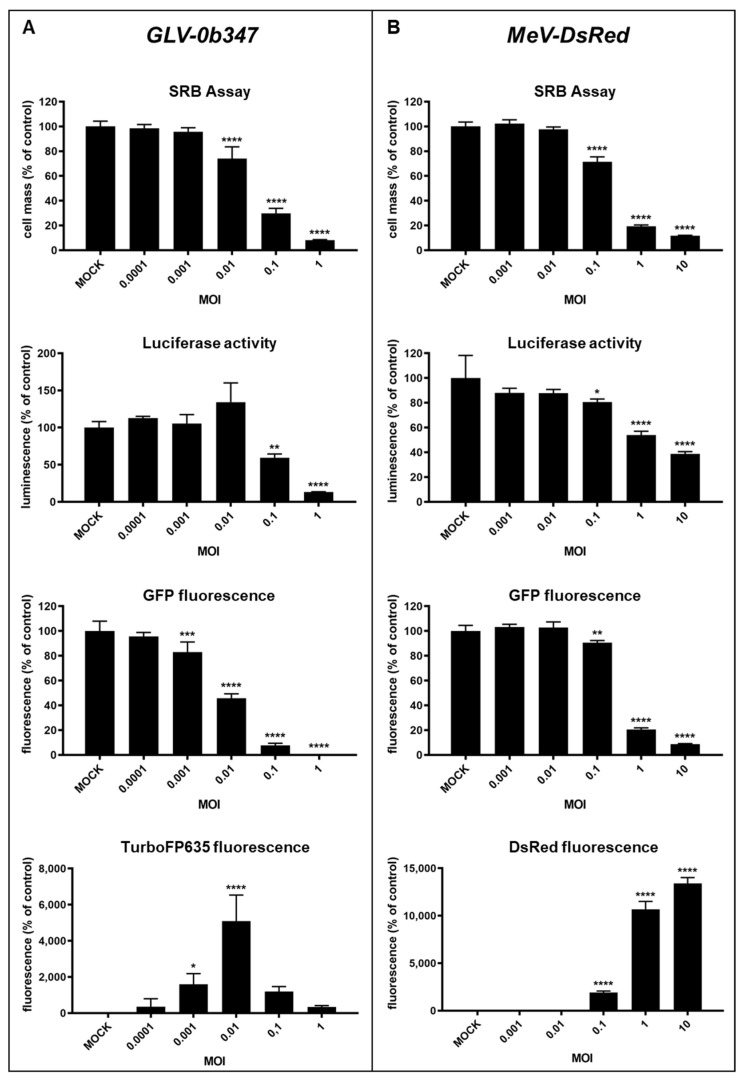
Comparison of different detection options for the oncolytic activity of virotherapeutic compounds GLV-0b347 (**A**, images to the left) and MeV-DsRed (**B**, images to the right) in HT-29 GFP/luc tumor cells. HT-29 GFP/luc cells were infected with GLV-0b347 (**A**) or MeV-DsRed (**B**) at different multiplicities of infection (MOIs) ranging from 0.0001 to 1 for GLV-0b347, from 0.001 to 10 for MeV-DsRed, or remained uninfected (MOCK). At 72 h postinfection (hpi), remaining tumor cell masses were determined by either (i) SRB viability assays, (ii) the measurement of the luciferase activity, or (iii) the quantification of the GFP or red-fluorescence intensity. Each measurement was calculated relative to the MOCK control. The mean ± SD of at least two independent experiments performed in triplicate is shown. ANOVA test relative to MOCK-infected control: * *p* < 0.05, ** *p* < 0.01, *** *p* < 0.001, and **** *p* < 0.0001.

For this purpose, GFP/luc-labeled human HT-29 colorectal cancer cells were infected in vitro with increasing MOIs of vaccinia virus GLV-0b347 (Figure 3A) or measles vaccine virus MeV-DsRed (Figure 3B). At 72 hpi, virus-mediated oncolysis was analyzed using (i) the SRB assay, (ii) the measurement of luciferase activity, and (iii) the quantification of GFP and TurboFP635/DsRed fluorescence (Figure 3). As expected, the quantification of GFP and TurboFP635 fluorescence signal in GLV-0b347-infected cells mirrored the observations in the fluorescence microscopy images. The greatest infection was seen with an MOI of 0.01, as reflected by the maximum TurboFP635 signals at 72 hpi, while GFP fluorescence decreased markedly when tumor cells were infected with MOIs higher than 0.01. Furthermore, the results of the SRB assay confirmed the successful MOI-dependent GLV-0b347-induced oncolysis of HT-29 cells with nearly identical sensitivity. This assay also showed that the remaining tumor cell masses decreased markedly at 72 hpi with GLV-0b347 at an MOI of 0.01 and higher. When luciferase activity was examined, a slight increase was observed at an MOI of 0.01, which may be explained by an initial virus-mediated boost in general cell metabolism [14,15]. However, at higher virus concentrations (MOIs of 0.1 and 1), the expected pronounced reduction in luciferase activity occurred, which again could be attributed to the significant oncolysis of HT-29 cells (Figure 3A).

In MeV-DsRed-infected cells, a nearly identical pattern of results for each detection method was observed (Figure 3B). The greatest infection was seen with MOIs of 1 and 10, as reflected by the maximum DsRed signals, while GFP fluorescence decreased markedly in these samples. The determination of both luciferase activity and remaining tumor cell mass with the SRB assay confirmed the data that had already been obtained by the measurement of GFP fluorescence, namely that the MeV-DsRed infection of HT-29 cells with increasing amounts of infectious virus particles resulted in a dose-dependent oncolysis of tumor cells (Figure 3B). 

In all the different assays tested, GLV-0b347 had a higher cell-killing capacity compared to MeV-DsRed, leading to higher oncolysis at lower MOIs. This is in accordance with previous studies, which showed that vaccinia viruses replicate faster and thus lead to almost complete cell death within a shorter time as compared to other oncolytic viruses such as measles viruses [16,17].

Notably, since an MOI-dependent increase in oncolysis of HT-29 cells was observed when applying both virus constructs, we believe that it is important to use sufficiently high virus concentrations for the study of ex vivo peritoneum co-culture models. Since all detection options showed almost comparable sensitivity in HT-29 cells alone, we decided to use the easy-to-perform luciferase measurements to determine treatment efficacies in the co-culture model.

### 3.3. Human Ex Vivo Peritoneum Model as a Platform for Virotherapeutic Applications

**Figure 4:**
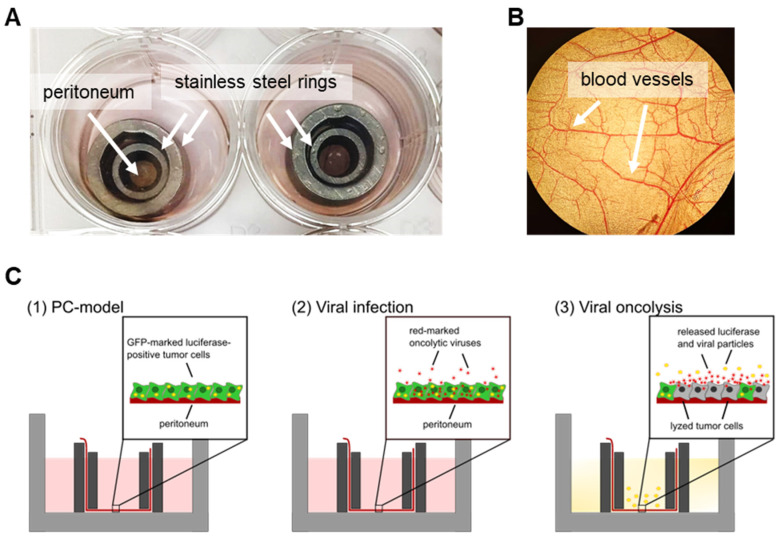
Human ex vivo peritoneum model and schematic illustration of the three-step virotherapeutic process in co-cultures with GFP/luc-labeled human HT-29 tumor cells. (**A**) Photographic image of the human ex vivo peritoneal model cultivated between stainless steel rings in a 24-well plate. (**B**) Photographic image of the peritoneum in the ex vivo model through a light microscope. (**C**) (1) Preparation of co-cultures of the peritoneum from noncancer patients and human GFP/luc-labeled HT-29 tumor cells. Successful plating of the cells can be verified by fluorescence microscopy. (2) Virotherapeutic treatment of co-cultures with oncolytic viruses carrying a red-fluorescent marker protein. Successful infection of the tumor cells can be verified by the determination of red fluorescence via fluorescence microscopy. (3) Viral oncolysis can be determined by a decrease in GFP and red fluorescence as well as by a decrease in luciferase activity.

**Figure 5:**
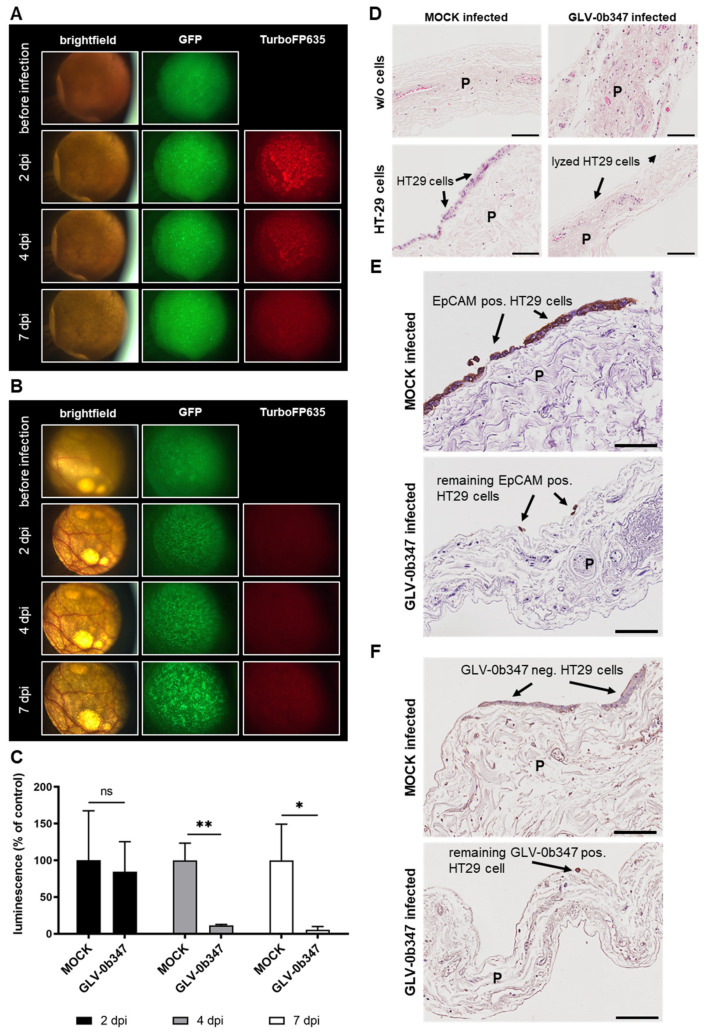
Virotherapeutic treatment of PC models with recombinant vaccinia virus GLV-0b347. The fluorescence images of GLV-0b347-infected co-cultures ((**A**); 1.5 × 10^6^ plaque-forming units (PFU)) or MOCK-infected (**B**) co-cultures consisting of the peritoneum of noncancer patients and adherently growing GFP/luc-labeled human HT-29 tumor cells at days 2, 4, and 7 postinfection (dpi); original magnification 4×. (**C**) Luciferase activity of GFP/luc–HT-29 cells growing on peritoneum at 2, 4, and 7 dpi, either GLV-0b347-infected or MOCK-infected. Each measurement is normalized to the MOCK control. The mean ± SD of one experiment performed in triplicates is shown. *t*-test relative to MOCK-infected control: * *p* < 0.05 and ** *p* < 0.01. ns; not significant. (**D**) The hematoxylin and eosin staining of human peritoneal tissue with and w/o co-culture of GFP/luc-labeled HT-29 cells at 7 dpi, either GLV-0b347-infected or MOCK-infected. (**E**) The EpCAM staining of peritoneal tissue with the co-culture of HT-29 cells at 7 dpi, either GLV-0b347-infected or MOCK-infected. (**F**) The vaccinia virus staining of peritoneal tissue with co-culture of HT-29 cells at 7 dpi, either GLV-0b347-infected or MOCK-infected. Experiments were conducted with the peritoneal tissue from different patients and show representative data from at least three different experiments. P; peritoneum. Black arrows indicate intact or infected and lysed HT-29 cells on the surface of the peritoneum. Scale bars represent 100 μm.

**Figure 6:**
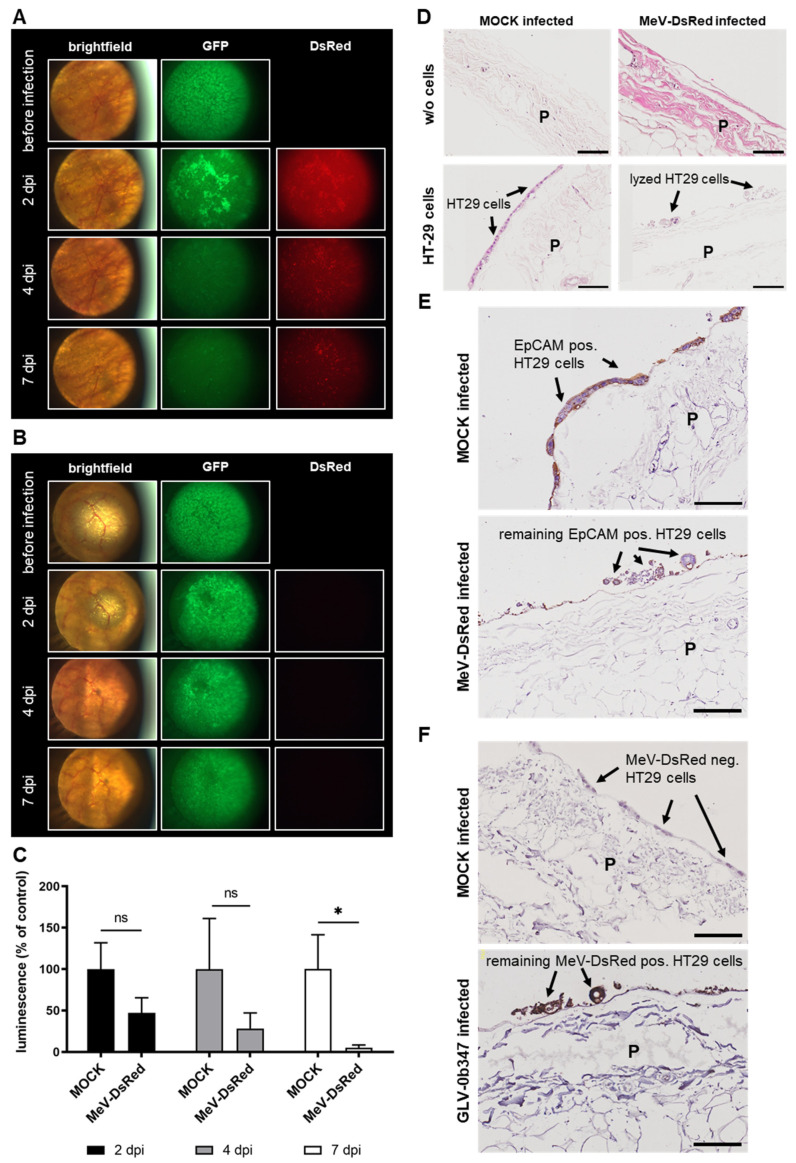
Virotherapeutic treatment of PC models with recombinant measles vaccine virus MeV-DsRed. The fluorescence images of MeV-DsRed-infected co-cultures ((**A**); 1.5 × 10^6^ plaque-forming units (PFU)) or MOCK-infected (**B**) co-cultures consisting of the peritoneum of noncancer patients and GFP/luc-labeled human HT-29 tumor cells at days 2, 4, and 7 postinfection (dpi); original magnification 4×. (**C**) The luciferase activity of GFP/luc–HT-29 cells growing on peritoneum at 2, 4, and 7 dpi, either MeV-DsRed-infected or MOCK-infected. Each measurement is normalized to the MOCK control. The mean ± SD of one experiment performed in triplicates is shown. *t*-test relative to MOCK-infected control: * *p* < 0.05. ns; not significant. (**D**) The hematoxylin and eosin staining of human peritoneal tissue with and w/o co-culture of GFP/luc-labeled HT-29 tumor cells at 7 dpi with MeV-DsRed or MOCK infection. (**E**) The EpCAM staining of peritoneal tissue with co-culture of HT-29 cells at 7 dpi with MeV-DsRed or MOCK infection. (**F**) The MeV staining of peritoneal tissue with co-culture of HT-29 cells at 7 dpi with MeV-DsRed or MOCK infection. The experiments were conducted with peritoneal tissue from different patients and show representative data from at least three different experiments. P; peritoneum. Black arrows indicate intact or infected and lysed HT-29 tumor cells on the surface of the peritoneum. Scale bars represent 100 μm.

In the next step, the virotherapeutic effectiveness of GLV-0b347 and MeV-DsRed was tested for the first time with normal/nonmalignant peritoneum obtained from noncancer patients co-cultured with GFP/luc-labeled HT-29 cancer cells. For this purpose, the peritoneum obtained from the surgery room was inserted immediately between stainless steel rings, as described before [6] (Figure 4A,B). Next, GFP/luc-HT-29 cancer cells were seeded onto the peritoneum and, after subsequent attachment, infected with the oncolytic viruses GLV-0b347 or MeV-DsRed at a fixed virus dosage of 1.5 × 10^6^ plaque-forming units (pfu) (Figure 4C). The successful growth of tumor cells on the peritoneum as well as their successful infection and oncolysis could be demonstrated by (i) fluorescence microscopy, (ii) luciferase measurements, and (iii) immunohistochemistry (IHC) (Figure 5 and Figure 6).

### 3.4. Virotherapeutic Treatment of PC Models with Recombinant Vaccinia Virus GLV-0b347

Infection of co-cultures with GLV-0b347 showed a significantly higher TurboFP635 signal (Figure 5A) compared to the MOCK-infected co-cultures (Figure 5B). This effect was observed in the infected sample as early as 2 dpi but subsequently decreased slightly over time. The GFP signal of tumor cells was stable in GLV-0b347-infected co-cultures but increased over time in MOCK-infected co-cultures. In addition, luciferase activity was clearly reduced in virus-infected co-cultures starting at 2 dpi, and further decreased strongly until 7 dpi compared to MOCK-treated samples, thereby indicating successful virus-induced oncolysis (Figure 5C). The IHC staining of co-cultures with GFP/luc-HT-29 revealed an effective tumor cell lysis and almost no remaining tumor cells in GLV-0b347-infected co-cultures compared to MOCK-treated co-cultures, as indicated by staining with hematoxylin and eosin (H&E) (Figure 5D) and EpCAM antibody (Figure 5E). Moreover, virus-specific stainings showed a selective infection of GFP/luc-HT-29 cells with GLV-0b347 without any signs of peritoneal infection (Figure 5F).

### 3.5. Virotherapeutic Treatment of PC Models with Recombinant Measles Vaccine Virus MeV-DsRed

When comparing MeV-DsRed-infected (Figure 6A) with MOCK-treated co-cultures (Figure 6B) under the fluorescence microscope, a significantly higher DsRed signal was observed in the infected sample as early as 2 dpi, which remained at a constant level until the end of the experiment at 7 dpi. The GFP signal of tumor cells obtained before infection was clearly visible with comparable intensities in both samples but decreased over time in the MeV-DsRed-treated co-culture. In addition, luciferase activity was clearly reduced in virus-infected co-cultures starting at 2 dpi, and further decreased until 7 dpi compared to MOCK-treated samples, thereby also indicating successful virus-induced oncolysis (Figure 6C). In addition, IHC staining was performed, which showed efficient tumor cell lysis and reduced numbers of tumor cells in MeV-DsRed-infected compared to MOCK-treated co-cultures, as indicated by staining with H&E (Figure 6D) and EpCAM antibody (Figure 6E). Moreover, virus-specific stainings revealed a selective infection of GFP/luc HT-29 tumor cells with MeV-DsRed without infection of normal cells constituting the nontumorous peritoneal tissue (Figure 6F). This tumor cell-restricted infection was also confirmed when infections were performed in ex vivo peritoneum models without the co-culture of HT-29 tumor cells (Supplementary Materials Figure S2). In ex vivo models without co-culture, neither infection with GLV-0b347 (Supplementary Materials Figure S2A) nor with MeV-DsRed (Supplementary Materials Figure S2B) resulted in the fluorescence microscopic detection of TurboFP635 or DsRed on the peritoneal tissue at 7 dpi. In contrast, both a distinct TurboFP635 (Supplementary Materials Figure S2A) and a pronounced DsRed fluorescence signal (Supplementary Materials Figure S2B) were observed at the same time point in peritoneum models with HT-29 tumor cells.

In summary, these results show that this platform could be highly valuable in testing patient- and tumor-specific responses to oncolytic virotherapy to determine the most effective virotherapeutic compound for each PC patient, thus paving the way for personalized virotherapy in PC. 

## 4. Discussion

Oncolytic virotherapy is a novel concept for the treatment of solid tumors that has expanded in recent years with a multitude of technology innovations directed at oncolytic viruses, showing promising results for several tumor entities [18,19,20]. However, most virotherapeutic compounds that are available today have only limited activity when given intravenously, which is attributed to (i) rapid clearance of the virus by the immune system before reaching the tumor, (ii) low infection rates of tumor sites when accessed by the blood circulation, and (iii) a low penetration depth into solid tumors.

PC is currently treated by cytoreductive surgery followed by intraperitoneal (i.p.) administration of chemotherapeutic agents (via pressurized intraperitoneal aerosol chemotherapy (PIPAC) or intraperitoneal hyperthermic chemoperfusion (HIPEC)). Since the peritoneal cavity can be reached quite easily, other possibilities for local treatment also exist, such as the direct intraperitoneal application of virotherapeutic compounds [21]. Thus, problems of blood-borne virus neutralization can be avoided (e.g., via the interaction with blood proteins and circulating antibodies). Moreover, it has been shown that aerosolization and hyperbaric pressure did not impact the suitability of adenoviruses when injected i.p., thereby allowing the possibility of using pressurized i.p. aerosolized virotherapy applications for the treatment of PC [22]. As a consequence, established clinical i.p. application methods such as PIPAC or HIPEC would be well suited for the i.p. application of novel therapeutics such as oncolytic viruses.

In line with this, Zhang et al. investigated the efficacy, safety, and immunomodulatory effect of i.p. injection of the oncolytic adenovirus H101 against malignant ascites in 40 patients. They showed that i.p. injection of the oncolytic virus H101 led to a marked tumor cell depletion without any major side effects. Moreover, H101 was found to mediate a tumor-specific immune activation on day 14 after treatment [23]. In addition, an ongoing phase III study is currently investigating the combination of an i.p. administered oncolytic vaccinia virus olvimulogene nanivacirepvec (Olvi-Vec; formerly known as GL-ONC1) in conjunction with platinum-doublet chemotherapy and bevacizumab in women diagnosed with platinum-resistant/ refractory ovarian cancer including primary peritoneal cancer (clinicaltrials.gov: NCT05281471). This phase III study builds on the promising results of a previous phase I study in which patients with platinum-resistant or refractory ovarian cancer were treated with the i.p. administration of Olvi-Vec as a monotherapy [24]. This trial demonstrated successful infection, in-patient replication, and subsequent oncolysis in the majority of patients during the first treatment cycle and defined a dosage of 3 × 10^9^ pfu/day i.p. on two consecutive days for phase II (RP2) trials. 

In a subsequent phase II trial (NCT02759588), the treatment regimen consisted of two consecutive days of the i.p. application of 3 × 10^9^ pfu Olvi-Vec followed by intravenous (i.v.) carboplatin-doublet (CD) ± bevacizumab (Bev). Thereafter, maintenance treatment was continued with single-agent therapies ± Bev. The mean number of applied cycles of CD ± Bev was determined as 6 (±3). The median follow-up was 26.5 months. RECIST ORR was 54% (95% CI: 33–74%) with 2 (8%) complete responses, 11 (46%) partial responses, and 8 (33%) stable diseases. The median duration of response was 7.6 months (95% CI: 3.7–9.6). The median PFS was 11.0 months (95% CI: 6.7–13.0), and the PFS-6-month was 77%. Thus, the majority of patients achieved RECIST response with a median PFS exceeding their prior line of therapy [25].

Other clinical trials in patients with PC from primary ovarian cancer and primary PC, but not primary colorectal cancer, are currently investigating the antitumor effect of different oncolytic viruses (measles [26], reovirus [27], vaccinia (NCT02759588), and adenovirus families (NCT00562003)) alone or in combination therapies. However, the oncolytic potential of these viruses in preclinical studies was mostly investigated in 2D-cultured cell lines. For example, the oncolytic herpes simplex viruses G207 and NV1020 were shown to successfully infect, replicate, and kill human gastric cancer cells in vitro and to reduce tumor burden in a murine xenograft model of peritoneally disseminated gastric cancer [28]. In another study, the oncolytic adenovirus OBP-401 synergistically suppressed the in vitro viability of human gastric cancer cells in combination with paclitaxel and inhibited the growth of peritoneal metastatic tumors and the number of malignant ascites in an orthotopic human gastric cancer peritoneal dissemination model [29]. Thus, these studies relied on 2D-cultured cell lines “only” and in in vivo mouse models (in part employing rather artificial xenograft tumor models), because meaningful 3D in vitro/ex vivo models for PC were not available at the time.

In our study, we have successfully established a human ex vivo peritoneum co-culture model for investigating and screening oncolytic virotherapeutics. In our state-of-the-art ex vivo setting, the GFP/luc-labeled human colorectal cancer cell line HT-29 grew well on patient-derived peritoneum and can be successfully infected and lysed by oncolytic vaccinia and measles vaccine viruses, as documented by microscopy and different analytical methods. In addition, oncolytic virotherapy was specific for HT-29 tumor cells without the noticeable infection of healthy human peritoneal tissue. Tumor specificity is a key prerequisite for the successful treatment of patients, as it reduces side effects and adverse events. 

Beyond that, our ex vivo PC model can be used for screening different oncolytic virotherapeutics in a clinically relevant model [7] using patient-derived primary cancer cells for co-culture [6] or patient-derived PC tissue. In this context, it is of the utmost importance to keep in mind that the immune system has a crucial influence on the efficacy of oncolytic virotherapy, which is why the immunological characterization of virotherapy in a possible autologous PC model provides promising insights in an application-oriented manner. Here, it would be of particular interest to assess whether immunogenic cell death (ICD) does occur, which is then accompanied by the release of proinflammatory cytokines and damage-associated molecular patterns (DAMPs), and whether IFN-stimulated genes (ISGs) are induced as a result of virotherapy. Furthermore, our new platform enables us to investigate the effects of co-cultured autologous PBMCs (either unstimulated or after stimulation with IL-2), NK cells, myeloid cells, and macrophages, and to determine whether these autologous cells play a significant tumor patient-individual role in combating PC. After the successful establishment of autologous immune cell applications, combination therapies with immune checkpoint inhibitors (e.g., pembrolizumab and atezolizumab) would also be conceivable, to investigate an enhanced synergistic antitumor effect. Taken together, our new platform offers multiple possibilities to obtain personalized data, which might predict the interplay of PC with immune cells in the respective tumor patients who are then treated with virotherapy based on our novel form of a companion diagnostic.

## 5. Conclusions

Here, we have successfully shown that a newly established human ex vivo PC model could be a suitable screening platform for testing various oncolytic virus constructs.

## Data Availability

The data presented in this study are available on request from the corresponding author.

## Supplementary Materials

The following supporting information can be downloaded at: https://www.mdpi.com/article/10.3390/v15020363/s1, Figure S1: Schematic structure of the used oncolytic viruses; Figure S2: Selective virotherapeutic infection of tumor cells in PC models.
