# Supplementary material for: Establishing a New Platform to Investigate the Efficacy of Oncolytic Virotherapy in a Human Ex Vivo Peritoneal Carcinomatosis Model
Source: Viruses. 2023 Jan 27;15(2):363. doi: 10.3390/v15020363 (PMC9963964; doi:10.3390/v15020363)
Supplement: Supplementary file 1 [file viruses-15-00363-s001.zip › Figure S1.pdf]

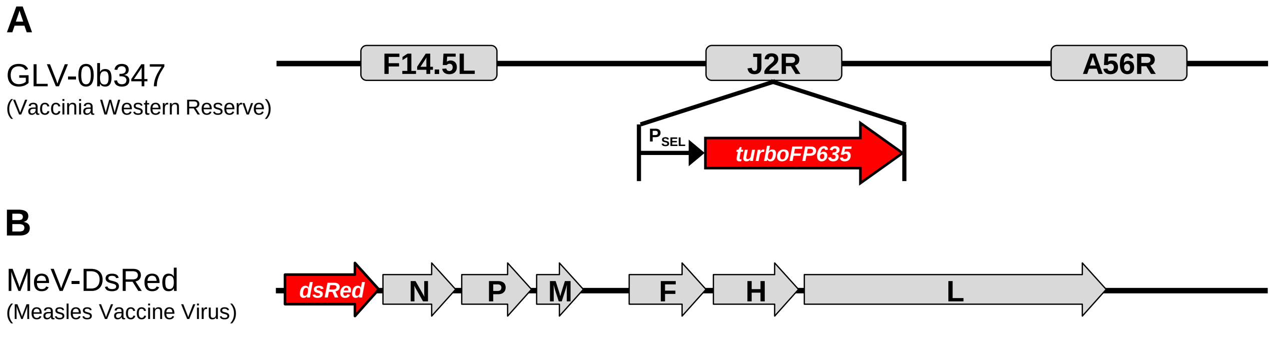

Figure S1. Schematic structure of the used oncolytic viruses. (A) The structure of the oncolytic vaccinia virus GLV-0b347 contains the red fluorescent marker gene for turboFP635 in the gene locus J2R under control of an early-late promotor. **(B)** The measles vaccinie virus MeV-DsRed contains the gene for the red fluorescent protein dsRed inserted before the N protein.
