# Supplementary material for: Establishing a New Platform to Investigate the Efficacy of Oncolytic Virotherapy in a Human Ex Vivo Peritoneal Carcinomatosis Model
Source: Viruses. 2023 Jan 27;15(2):363. doi: 10.3390/v15020363 (PMC9963964; doi:10.3390/v15020363)
Supplement: Supplementary file 1 [file viruses-15-00363-s001.zip › Figure S2.pdf]

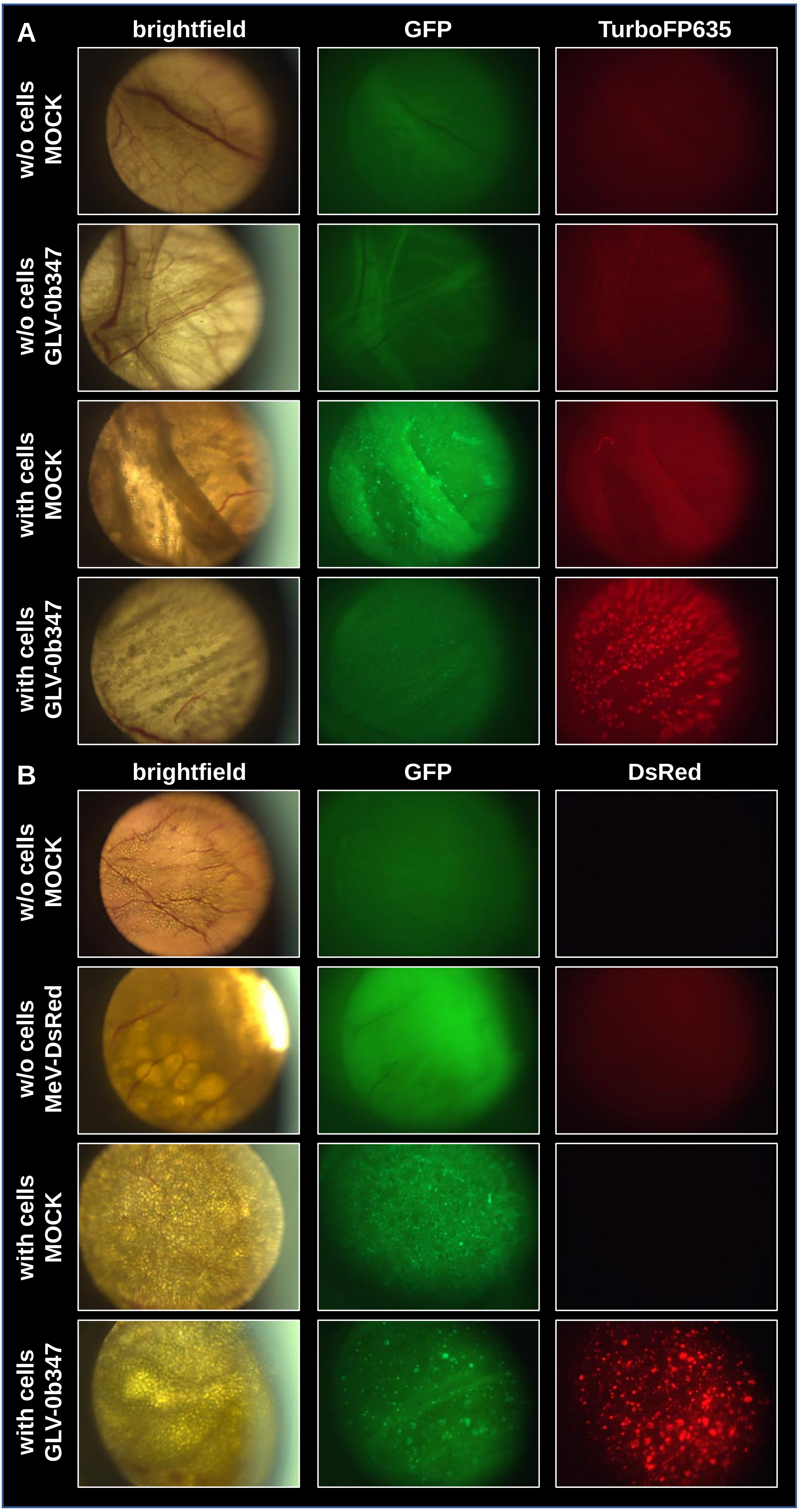

Figure S2. Selective virotherapeutic infection of tumor cells in PC models. (A) Fluorescence images of ex-vivo peritoneal models 7 days post-infection (dpi) with GLV-0b347 (1.5 x 107 PFU (plaque forming units) consisting of all controls including peritoneum of NON-cancer patients without (w/o) tumor cells as well as peritoneum with tumor cells, each infected and MOCK infected. **(B)** Fluorescence images of co-cultures 7 days post-infection (dpi) with MeV-DsRed (1.5 x 107 PFU (plaque forming units) consisting of all controls including peritoneum of NON-cancer patients without (w/o) tumor cells as well as peritoneum with tumor cells, each infected and MOCK infected. Original magnification 4x.
